# Instantaneous wave-free ratio derived from coronary computed tomography angiography in evaluation of ischemia-causing coronary stenosis: Feasibility and initial clinical research

**DOI:** 10.1097/MD.0000000000005979

**Published:** 2017-01-27

**Authors:** Yue Ma, Hui Liu, Yang Hou, Aike Qiao, Yingying Hou, Qingqing Yang, Qiyong Guo

**Affiliations:** a Department of Radiology, Shengjing Hospital of China Medical University, Shenyang; b Department of Radiology, Guangdong Academy of Medical Sciences, Guangdong General Hospital, Guangzhou; c College of Life Science and Bioengineering, Beijing University of Technology, Beijing, China.

**Keywords:** computational fluid dynamics (CFD), computed tomography angiography (CTA), coronary stenosis, fractional flow reserve (FFR), instantaneous wave-free ratio (iFR), instantaneous wave-free ratio derived from coronary computed tomography angiography (iFR_CT_)

## Abstract

The instantaneous wave-free ratio (iFR) closely related to fractional flow reserve (FFR) is a adenosine-independent physiologic index of coronary stenosis severity. We sought to evaluate whether iFR derived from coronary computed tomographic angiography (iFR_CT_) can be used as a novel noninvasive method for diagnosis of ischemia-causing coronary stenosis.

We retrospectively enrolled 33 patients (47 lesions) with coronary artery disease (CAD) and examined with coronary computed tomographic angiography (CTA), invasive coronary angiography (ICA), and FFR. Patient-specific anatomical model of the coronary artery was built by original resting end-diastolic CTA images. Based on the model and computational fluid dynamics, individual boundary conditions were set to calculate iFR_CT_ as the mean pressure distal to the stenosis divided by the mean aortic pressure during the diastolic wave-free period of rest state. Ischemia was assessed by an FFR of up to 0.8, while anatomically obstructive CAD was defined by a stenosis of at least 50% by ICA. The correlation between iFR_CT_ and FFR was evaluated. The receiver operating characteristic (ROC) curve was used to select the cut-off value of iFR_CT_ for diagnosis of ischemia-causing stenosis. The diagnostic performances of iFR_CT_, coronary CTA, and iFR_CT_ plus CTA for ischemia-causing stenosis were compared with ROC curve and Delong method.

On a per-vessel basis, iFR_CT_ and FFR had linear correlation (*r* = 0.75, *p* < 0.01). ROC analysis identified an optimal iFR_CT_ cut-off value of 0.82 for categorization based on an FFR cut-off value 0.8, and the diagnostic accuracy, sensitivity, specificity, positive predictive value (PPV), and negative predictive value (NPV) of iFR_CT_ were 78.72%,70.59%, 83.33%,70.59%, and 83.33%, respectively. Compared with obstructive CAD diagnosed by coronary CTA (AUC = 0.60), iFR_CT_ yielded diagnostic improvement over stenosis assessment with AUC increasing from 0.6 by CTA to 0.87 (*P* < 0.01) and 0.90 (*P* < 0.01) when iFR_CT_ plus CTA.

In conclusion, iFR_CT_ is a promising index improving diagnostic performance over coronary CTA for detection of ischemia-causing coronary stenosis.

## 1 Introduction

In recent years, coronary computed tomography angiography (CTA) has been proved to be a favorable means of morphological examination for coronary artery stenosis.^[1–4]^ However, simple morphological stenosis cannot reliably assess the functional significance of coronary heart disease (CHD).^[5–7]^ Comprehensive evaluation of coronary artery stenosis should include both morphological and functional aspects. Invasively measured fractional flow reserve (FFR) is widely recognized as the gold standard in evaluating the functional changes of coronary stenosis.^[8–10]^ Revascularization therapy based on FFR is a reliable method for improving prognosis and reducing medical costs, and it has been included in the guidelines as the gold standard for qualitative diagnosis of obstructive CHD and treatment strategy. However, in practice, the application of FFR is limited due to its invasive nature, and some patients find it hard to accept vasodilatory drugs and the high cost.^[11,12]^

Recently, a new derivative pressure indicator (instantaneous wave-free ratio (iFR)) in the resting state has been introduced, which can be measured without administration of vasodilatory drugs. iFR is defined as the ratio between the mean instant pressure distal to coronary stenosis and the mean pressure at the aortic root during wave-free period. The internal resistance of coronary artery is the lowest and most constant during wave-free period (defined as the diastolic window), which ranges from 25% of the way into diastole to 5 ms before its ending (close to 0). This is similar to the internal resistance of coronary artery achieved by vasodilator drugs during direct measurement of FFR.^[13]^ The ADVISE study reported that iFR had a good correlation with FFR (*r* = 0.9, *P* < 0.001) and showed excellent diagnostic efficiency. When FFR < 0.8, the area under the receiver operating characteristic curve (AUC) of iFR amounts to 0.93.^[13]^ The JUSTIFY-CFR study showed that iFR had a better correlation with coronary flow velocity reserve (CFVR) than FFR, which could be used in functional tests of vascular lesions independent of FFR.^[14]^ Also, van de Hoef et al^[15]^ investigated iFR and FFR against a combined reference of myocardial perfusion scintigraphy and hyperemic stenosis resistance (HSR), and they found that among 19% of the cases where iFR disagrees with FFR, FFR did not always produce highly accurate results, indicating that FFR is not necessarily a better discriminator of coronary ischemia than iFR. Moreover, iFR avoids the administration of vasodilatory drugs needed for FFR, and it has less variability and fewer repeated measurement differences compared with FFR (−0.0005 ± 0.002 vs 0.01 ± 0.04).^[13]^

So far, FFR derived from coronary CTA (FFR_CT_) has been used for integrated evaluation of anatomical and physiological functions of coronary stenosis, and it showed that the detection of hemodynamically significant stenosis evaluated by FFR_CT_ correlated well with invasive FFR.^[16–19]^ However, the application of iFR derived from coronary CTA (iFR_CT_) for hemodynamic assessment of coronary stenosis has not yet been studied. Therefore, the purpose of our study was to evaluate the correlation between iFR_CT_ and FFR, explore the diagnostic efficacy of iFR_CT_ in the diagnosis of functional ischemia, and investigate the clinical value of iFR_CT_ combined with CTA in the evaluation of coronary stenosis.

## 2 Methods

### 2.1 Study population

Clinical data of 33 patients with suspected or known CHD from August 2011 to December 2014 in 5 Chinese hospitals (Shengjing Hospital of China Medical University, the First Affiliated Hospital of China Medical University, General Hospital of Shenyang Military Command, Dalian Central Hospital, and General Hospital of Guangdong Province) were retrospectively collected. Inclusion criteria: patients who underwent coronary CTA, invasive coronary angiography (ICA), and FFR in a nonemergent setting. Exclusion criteria: time between procedures exceeding 2 months, major interprocedural adverse cardiac events (myocardial infarction, cardiac death, or revascularization), significant decrease of left ventricular function, complicated congenital heart diseases, previous coronary artery bypass surgery or stenting, installed pacemaker, artificial heart valves, bifurcation stenosis, chronic total occlusion, nondiagnostic quality of CTA data, and body mass index (BMI)≥35. The study protocol conformed to the ethical guidelines of the 1975 Declaration of Helsinki. The study was approved by the ethics committee of Shengjing Hospital of China Medical University, the ethics committee of the First Affiliated Hospital of China Medical University, the ethics committee of General Hospital of Shenyang Military Command, the ethics committee of Dalian Central Hospital, and the ethics committee of General Hospital of Guangdong Province. Informed consent from patients was not needed because of the retrospective nature of this study.

### 2.2 Image acquisition and analysis for CT

All the CTA scanning was carried out in accordance with the guidelines recommended by the Society of Cardiovascular Computed Tomography.^[20,21]^ CTA was performed with ≥64-slice multidetector scanners (Brilliance iCT 256, Philips Healthcare, Surrey, UK; Somatom Definition, Siemens, Forchheim, Germany; Aquilion One, Toshiba, Otawara, Japan; Optima CT660, GE Healthcare, Milwaukee, WI). Patients with heart rate >70 bpm were treated with (β)-blockers, and no nitroglycerin was given to any patient before CT examination. During the scanning, 60–80 Ml contrast agent (Omnipaque, 350 mgI/mL, GE Healthcare; Visipaque 320 mg/dL, GE Healthcare; Iopromide, 370 mgI/mL, Bayer) was administrated through intravenous injection (4–5 mL/s), followed by rinsing with 20 to 30 mL saline. Retrospective electrocardiography (ECG)-gated spiral scanning or prospective ECG-triggering axis scanning was used. Scanning parameters included: collimator width of (2 × 64)/128/320 × 0.5/0.625 mm; 100 or 120 kV; effective tube current of 400 to 700 mA. Effective dose (ED) for CTA was calculated using the following formula: ED (mSv) = (dose length product) × 0.014. The effective radiation dose was 1.5–10.8 mSv.

The original data were reconstructed using standard convolution function, with a sharp convolution function reconstruction algorithm used for cases with severe coronary artery calcification. The best quality end-diastolic phase images were selected for further analysis.

Radiologists with 7 years of experience or more evaluated image quality and analyzed coronary stenosis. The image quality of coronary CTA was assessed using a 4-point Likert scale (1 point = poor image quality, nondiagnostic; 2 points = satisfactory, reduced image quality caused by motion artifacts, image noise or limited luminal contrast, but was good enough for luminal evaluation; 3 points = good vessel contrast without major artifacts; 4 points = excellent, no diagnostic limitations).^[22]^ Images with quality scores of 2 to 4 points were included in the analysis. The evaluation of stenosis was carried out using blood vessels as the unit, which consisted of the left anterior descending artery (including first and second diagonal branches), left circumflex artery (including the middle branch), and right coronary artery (including the right posterior and posterior descending branches). Stenosis was classified as 4 grades through visual inspection: mild stenosis (lumen diameter reduced by <50%), moderate stenosis (lumen diameter reduced by 50%–69%), severe stenosis (lumen diameter reduced by 70%–98%), and subtotal-to-total occlusion (lumen diameter reduced by 99%–100%). Per-vessel stenosis severity was evaluated by the extent of the maximal stenosis (lesion of interest) within the vessel.

Coronary plaques were defined as visible structures >1.0 mm^2^ located on the vascular wall or in the surrounding lumen, which could be clearly distinguished from epicardial adipose tissues in the lumen or surrounding tissues. The CT value of calcified components was >130 HU, and that of noncalcified components was <130 HU. According to plaque composition, the coronary lesions of interest were classified as a significantly calcified plaque group (>70% of the plaque volume was occupied by calcified components), or a nonsignificantly calcified plaque group (≤70% of the plaque area was occupied by calcified components).^[23]^

### 2.3 Image acquisition and analysis for ICA

ICA was performed by an experienced interventional cardiologist. A reference guide tube was used for correction, along with an edge detection system for measuring the reference diameter, minimum luminal diameter, length of the lesion, and calculation of the percentage of stenosis. The stenosis was divided into 4 grades as follows: mild stenosis (stenosis percentage <50%), moderate stenosis (50%–69%), severe stenosis (70%–98%), and subtotal-to-total occlusion (99%–100%). Evaluation of thrombolysis in myocardial infarction flow grades during imaging was as follows: Grade 0, no blood perfusion or blood flow at the distal end of the coronary artery; Grade 1, some contrast agent reached the distal end of the coronary stenosis with incomplete filling; Grade 2: the distal end of the coronary artery stenosis could be completely filled, but the image development and elimination of contrast agent were slow; Grade 3: complete and rapid filling and elimination of contrast agent at the distal end of the coronary artery; similar to normal coronary arteries.

### 2.4 Invasive measurement of FFR

In case of significant clinical symptoms or ≥50% stenosis was observed on the ICA results, FFR was measured (Pressure Wire Certus, St Jude Medical Systems, Uppsala, Sweden; Combo Wire, Volcano Corporation, San Diego, CA). Prior to measurement, nitroglycerin (100–200 g) was administered into the coronary artery, and then a pressure monitoring guide wire was delivered to the distal end of the stenosis, followed by intravenous or intracoronary injection of adenosine triphosphate (ATP) (140 g/kg/min) to induce a maximum congestive state. FFR was equal to the ratio of mean distal stenosis pressure to mean aortic root pressure at the maximal coronary hyperemia status. FFR≤0.80 was specified as the diagnostic standard for functional stenosis.^[10]^

### 2.5 Calculation of iFR_CT_

iFR_CT_ blood flow dynamics modeling and computational fluid dynamics numerical simulation were carried out by an independent core laboratory (Biomedical Engineering Laboratory of Beijing University of Technology, Beijing, China) blinded to FFR, coronary CTA, and ICA results. The 3 key steps were as follows: first, based on the DICOM image of conventional coronary CTA at the end-diastolic stage, Mimics 10.1 (Materialise, Leuven, Belgium), Geomagic Studio 2014 (Geomagic Inc, Cary, NC) and SolidWorks 2014 (Dassault Systèmes Inc, SOLIDWORKS Corp, Waltham, MA) were used to establish an accurate individualized anatomical model of the coronary artery. Second, based on the principle that resting coronary blood flow is proportional to myocardial oxygen demand, the individual boundary conditions at the entrance and exit of the coronary artery were calculated according to the myocardial mass and mean arterial pressure of individual patients. Third, the fluid dynamics Navier Stokes equation (ANSYS Workbench 14.5, ANSYS Inc, Canonsburg, PA) was used for iterative calculation, extraction, analysis, and visualization. The pressure difference (Δ*P*) between each point of the coronary artery lesions and the entrance of the coronary artery was calculated. iFR_CT_ was defined as the mean pressure distal to the stenosis during the diastolic wave-free period of rest state (Pd_wave-free period of rest state_) divided by the mean aortic pressure during the same state (Pa_wave-free period of rest state_) (Equation 1), in which Pd_wave-free period of rest state_ was equal to Pa_wave-free period of rest state_minus ΔP (Equation 2), and thus the contours of iFR_CT_ were obtained. The whole process of modeling and calculation for each case took about 4 hours. 
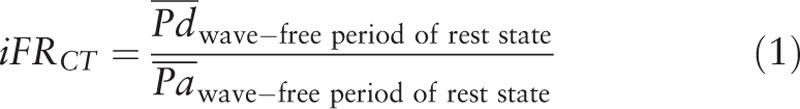
 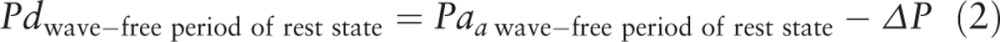


### 2.6 Statistical analyses

SPSS for Windows version 20.0 (SPSS, Chicago, IL) and MedCalc for Windows version 12.7.7 (MedCalc Software, Ostend, Belgium) were used for statistical analysis. All variable data were presented as mean ± standard deviation. Pearson correlation analysis was used to analyze the correlation between FFR and iFR_CT_, thus comparing the correlation difference of FFR and iFR_CT_ between the significantly and nonsignificantly calcified plaque groups. FFR value was used as the gold standard for diagnosis of functional coronary stenosis. The ROC curve was used to select the most suitable cut-off value of iFR_CT_ for the diagnosis of functional coronary artery stenosis, and to investigate the diagnostic efficiency of iFR_CT_ for functional stenosis. The difference between the diagnostic efficiency of iFR_CT_ in the significantly and nonsignificantly calcified plaque groups was evaluated using Fisher test. Coronary CTA luminal stenosis ≥50% was determined as the critical value for the diagnosis of functional stenosis-causing myocardial ischemia. Logistic regression was used to calculate the predictive probability of CTA combined with iFR_CT_, and then ROC curve analysis was used to investigate the impact of coronary CTA combined with iFR_CT_ on diagnostic efficiency for functional stenosis. The difference of AUC in the diagnosis of functional stenosis using CTA alone and CTA combined with iFR_CT_ was compared using Delong method. *P*<0.05 was considered statistically significant.

## 3 Results

### 3.1 Characteristics of study subjects

This study included 33 eligible patients with a total of 47 vascular lesions. The demographic and clinical characteristics of the study population are shown in Table 1. The analysis was based on each vessel, and the results of ICA, CTA, and FFR for all 47 lesions are listed in Table 2.

**Table 1:**
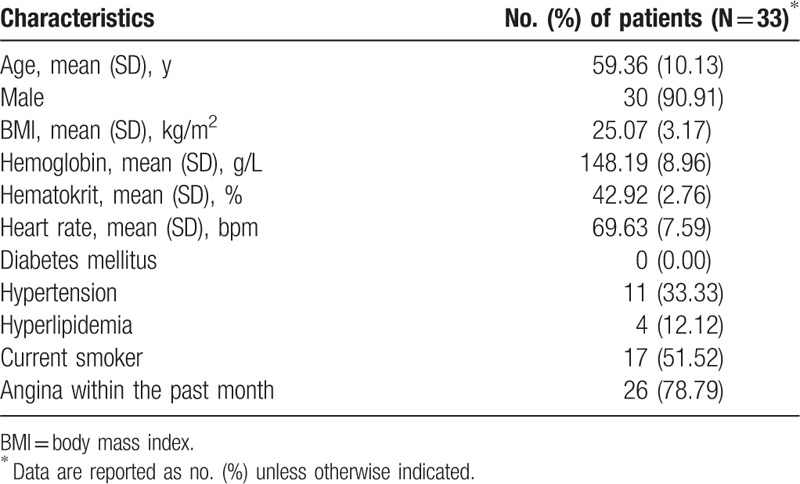
Baseline characteristics of the study population.

**Table 2:**
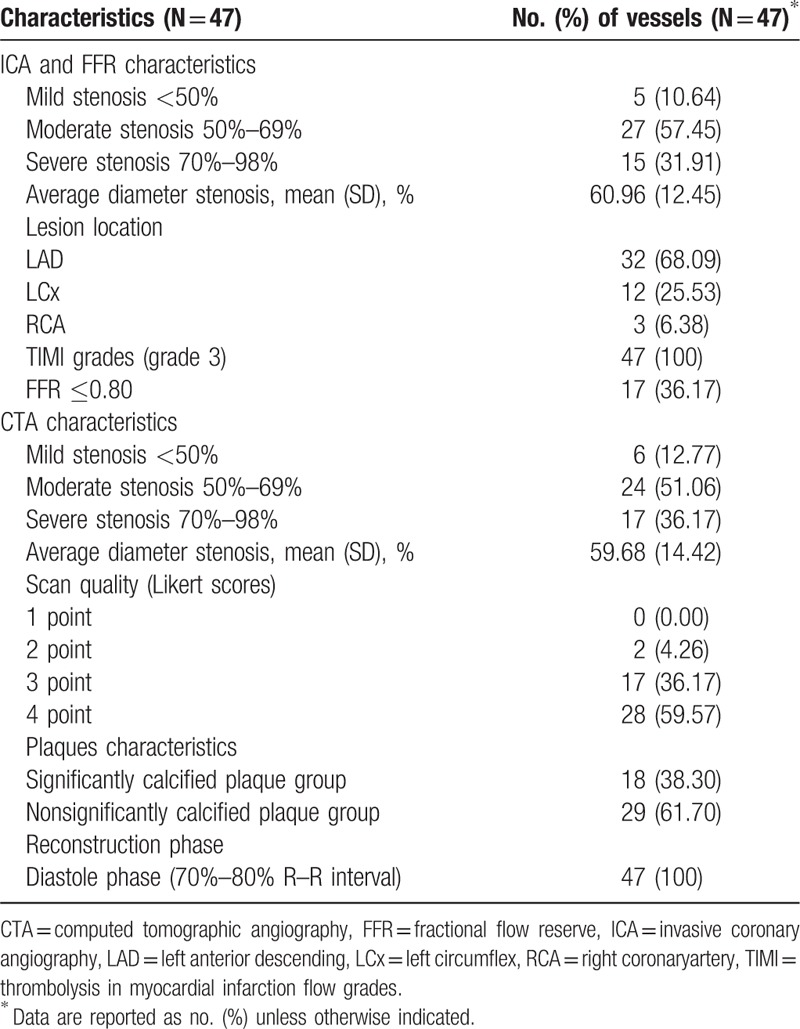
Vessels characteristics by ICA, FFR, and CTA.

### 3.2 Correlation between iFR_CT_ and FFR

**Figure 1:**
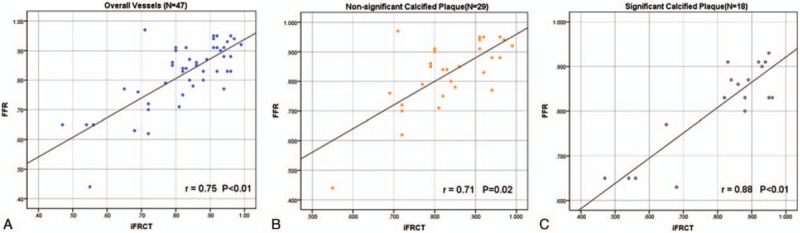
Correlation of iFR_CT_ and invasive FFR. A strong correlation was observed between iFR_CT_ and FFR in overall vessels (A), the group of nonsignificant calcified plaque (B), and the group of significant calcified plaque (C). FFR = fractional flow reserve, iFR_CT_ = instantaneous wave-free ratio derived from coronary computed tomography angiography.

**Figure 2:**
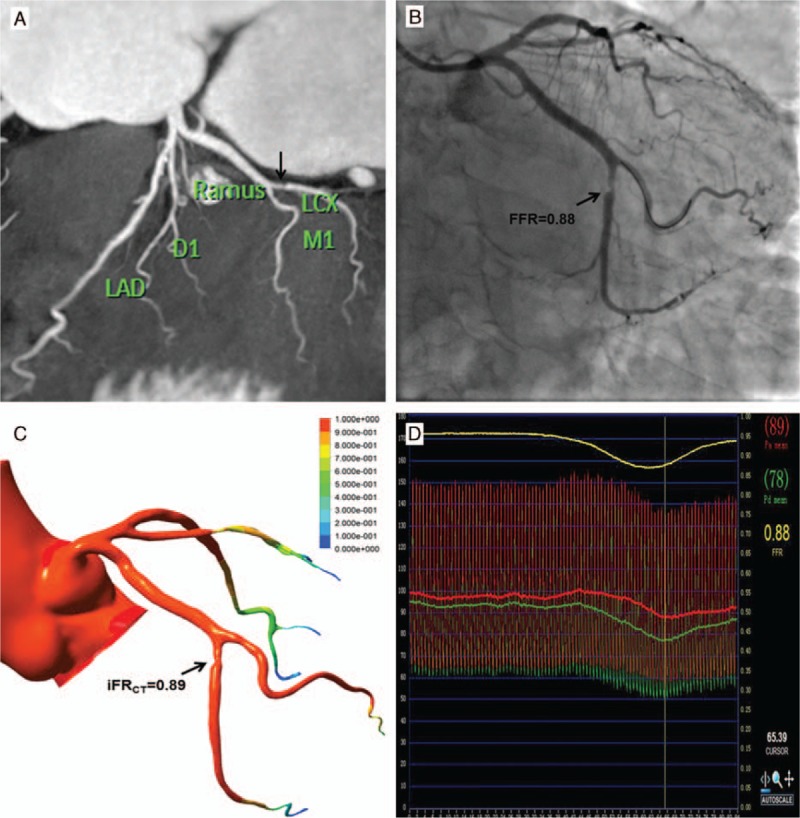
Coronary CTA, displayed as 2D with maximum intensity projection reformation of the left coronary artery, showed a moderate luminal stenosis (50–69%) in the mid portion of the LCX vessel (arrow) (A). Invasive coronary angiography confirmed the lesion (B). Catheter-based FFR of the moderate stenosis lesion of LCX was measured at 0.88, indicating lack of hemodynamic significance of this lesion (D). Noninvasive iFR_CT_ resulted in a value of 0.89 for the lesion of LCX (arrow), in good correlation with invasive measurement (C). CTA = computed tomography angiography, FFR = fractional flow reserve, LCX = left circumflex.

For all blood vessels, the iFR_CT_ value was higher than the FFR value. However, the iFR_CT_ value of the significantly calcified plaque group was lower than the FFR value (Table 3). There was a significant linear correlation between FFR and iFR_CT_ (Fig. 1). There was no significant difference in the correlation of FFR and iFR_CT_ between the significantly and nonsignificantly calcified plaque groups (95% confidence interval (CI) 0.72–0.95 vs 0.32–0.90, respectively) (Table 3). Representative example of anatomically obstructive stenosis without ischemia-producing stenosis is shown in Fig. 2.

**Table 3:**
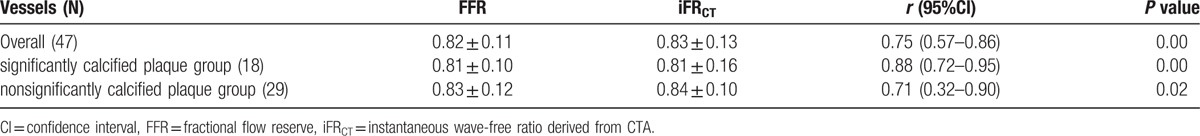
Correlation of iFR_CT_ and invasive FFR.

### 3.3 The optimal cut-off value and diagnostic efficiency of iFR_CT_ in the diagnosis of coronary stenosis

**Figure 3:**
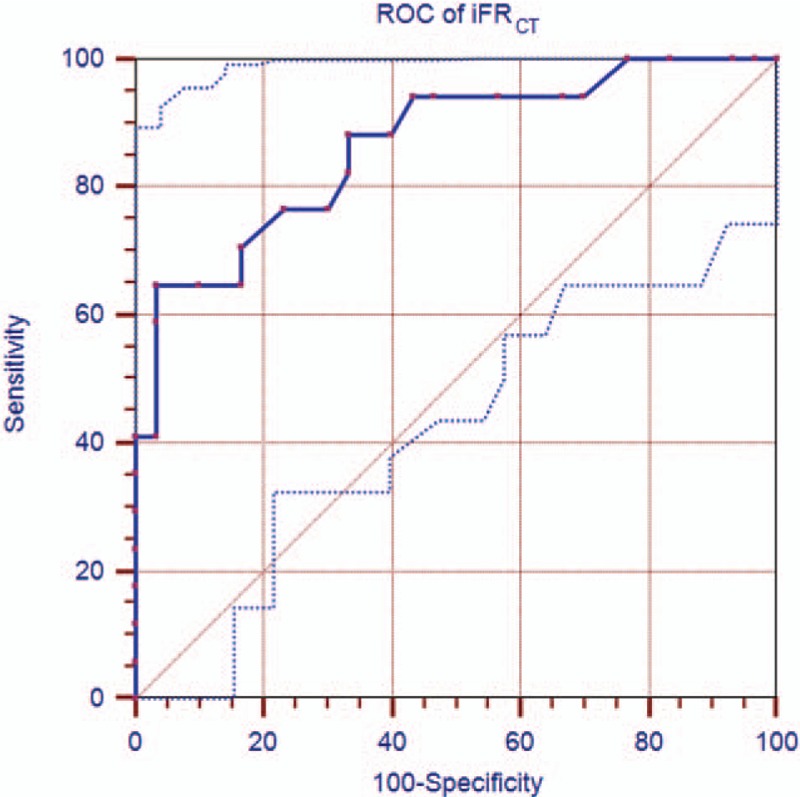
Receiver operating characteristic curve of per-vessel performance of iFR_CT_ compared with invasive FFR≤0.80 for diagnosis of ischemia.

FFR≤0.80 was determined as the gold standard for the diagnosis of functional stenosis-causing myocardial ischemia. AUC for iFR_CT_ was 0.87 (95% CI 0.75–0.98, *P* < 0.01), with an optimal iFR_CT_ cut-off value of 0.82 (Fig. 3). At this point, the diagnostic accuracy of iFR_CT_ for coronary stenosis was 78.72%, with sensitivity, specificity, positive predictive value (PPV), and negative predictive value (NPV) of 70.59%, 83.33%, 70.59%, and 83.33%, respectively.

### 3.4 Comparison of iFR_CT_ diagnostic efficiency between significantly and nonsignificantly calcified plaque groups

There were 18 vascular lesions in the significantly calcified plaque group, of which lesions with FFR ≤0.80 accounted for 33.33% (n = 6). The diagnostic accuracy, sensitivity, specificity, PPV, and NPV of iFR_CT_ in this group were 94.44% and 83.33%, 100%, 100%, and 92.31%, respectively. There were 29 vascular lesions in the nonsignificantly calcified plaque group, of which lesions with FFR ≤0.80 accounted for 37.93% (n = 11). The diagnostic accuracy, sensitivity, specificity, PPV, and NPV of iFR_CT_ in this group were 68.97%, 63.64%, 72.22%, 58.33%, and 76.47%, respectively. However, there was no significant difference in the diagnostic accuracy of iFR_CT_ between these 2 groups detected by Fisher exact test (*P* = 0.07).

### 3.5 Efficacy of CTA combined with iFR_CT_ in the diagnosis of functional coronary stenosis

**Figure 4:**
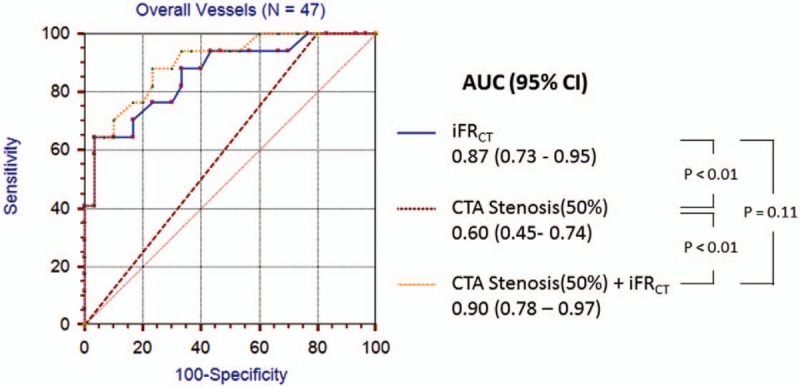
Receiver operating characteristic curve demonstrating AUC for iFR_CT_, CTA stenosis, and the combination of the both for discrimination of lesions that caused ischemia on a per-vessel level. The AUC for iFR_CT_ and the combination of the both were significantly higher than CTA stenosis.

FFR≤0.80 was determined as the gold standard for the diagnosis of ischemic stenosis. The efficacy of coronary CTA and iFR_CT_ alone, and their combination in the diagnosis of ischemic stenosis is shown in Fig. 4 and Table 4. The diagnostic accuracy of CTA (50%) was increased to 82.98% when introducing iFR_CT_, with its specificity increased to 90% and PPV to 80%. Compared with the AUC when using coronary CTA alone for diagnosis, the diagnostic efficacy of CTA (50%) was significantly higher after introduction of iFR_CT_ (0.6 vs 0.9, *P* < 0.01), with the AUC value of the ROC curve of 0.90, demonstrating that diagnosis of coronary ischemic stenosis was significantly improved by combining CTA with iFR_CT_ (*P* = 0.04).

**Table 4:**
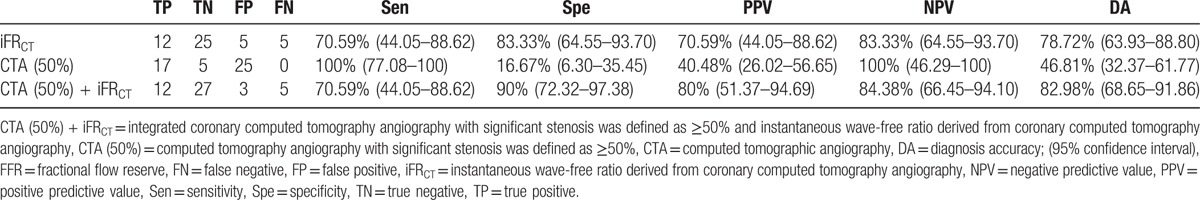
Diagnostic performance of iFR_CT_, CTA stenosis, and CTA stenosis combined with iFR_CT_ on a per-vessel basis, using invasive FFR as the reference standardin diagnosis of functional coronary stenosis.

## 4 Discussion

The current study demonstrated a good correlation between iFR_CT_ and FFR. The application of iFR_CT_ showed high efficiency for the diagnosis of coronary stenosis, and it also had good diagnostic accuracy for functional stenosis caused by significantly calcified plaques. More importantly, using FFR≤0.80 as the gold standard for control, the diagnostic ability of iFR_CT_ combined with coronary CTA for functional stenosis was significantly improved, with higher specificity and PPV compared with using CTA alone.

To the best of our knowledge, this study is the first blood flow dynamics simulation and calculation of resting state index iFR based on conventional coronary CTA images. Sen et al^[13]^ found a transient wave-free interval during cardiac diastole (from 25% of the process of diastole to 5 ms before its ending), during which time the measured cardiac microcirculation resistance was naturally constant and minor. This is similar to the average microcirculation resistance of the whole cardiac cycle during maximum cardiac expansion induced by adenosine.^[13]^ iFR is in theory similar to FFR and its result is independent of heart rate, blood pressure, and even blood pressure change caused by ectopic arrhythmia or respiration.^[13]^ The ADVISE study showed that iFR had good reproducibility (*r* = 0.996, *P* < 0.01) and was highly correlated with FFR (*r* = 0.90).^[13]^ However, the VERIFY study drew different conclusions, pointing out that iFR was influenced by vasodilators and correlated weakly with FFR.^[24]^ In addition, the RESOLVE study and Johnson et al found hyperemic indicators provided diagnostic performance superior to that of resting iFR.^[25,26]^ Although resistance was reduced further by administration of adenosine, the CLARIFFY study demonstrated that differences in magnitude of microvascular resistance did not influence diagnostic categorization, and iFR, iFRa (adenosine administration), and FFR had equally good diagnostic agreement with HSR.^[27]^ Meanwhile, Petraco et al proved that hyperemic FFR flow was similar to baseline iFR flow in functionally significant lesions (FFR ≤0.75; mean FFR flow, 25.8 ± 13.7 cm/s vs mean iFR flow, 21.5 ± 11.7 cm/s; *P* = 0.13).^[14]^ Furthermore, iFR showed a stronger correlation with underlying CFVR (iFR–CFVR, ρ=0.68 vs FFR–CFVR, ρ=0.50; *P* < 0.001) and also agreed more closely with CFVR in stenosis classification (iFR AUC = 0.82 vs FFR AUC = 0.72; *P* < 0.001, for a CFVR of 2).^[14]^ Therefore, iFR can be used as an adenosine-free alternative for FFR with a good diagnostic performance. When 0.83 is used as the threshold for iFR, its diagnostic sensitivity is 91%, with specificity of 85%, PPV of 91%, and NPV of 85%.^[13]^ The ADVISE registry study demonstrated that, based on the inherent variability of FFR, the consistency in critical lesion classification using iFR≤0.89 and FFR≤0.80 as the threshold was as high as 94%, and 81% of the critical lesions with inconsistent classification had associated FFR values located within the FFR “gray zone”(0.75–0.80).^[28]^ Petraco et al^[29]^ used iFR < 0.86 as the threshold (PPV 92%) for percutaneous coronary intervention (PCI), iFR>0.93 as the threshold (NPV 91%) for delay of PCI, and only performed adenosine detection of FFR in patients with iFR0.86–0.93. Such an iFR–FFR hybrid method can reduce the use of adenosine in patients by 57%, and achieve a classification consistency of 95% with FFR.^[29]^ The results of the current study show that iFR_CT_ is significant correlated with the invasively measured FFR, just as in previous studies,^[13,24,25,27,28,30]^ which provides a new evidence supporting the use of iFR as an evaluation index for functional stenosis.

Our study demonstrated the high efficiency of using iFR_CT_ for the diagnosis of coronary artery stenosis. The diagnostic accuracy, sensitivity, specificity, PPV, and NPV were 78.72%, 70.59%, 83.33%, 70.59%, and 83.33%, respectively. Such results are mostly equivalent to the diagnostic efficacy of FFR_CT_ reported in previous multicenter studies. Previous prospective studies, DISCOVER-FLOW,^[16]^ DeFACTO,^[31]^ and NXT^[32]^ have demonstrated diagnostic accuracy, sensitivity, specificity, PPV, and NPV of FFR_CT_ of 73% to 81%, 86% to 90%, 54% to 82%, 65% to 74%, and 84% to 93%, respectively. Our study confirmed the feasibility of using a computational fluid dynamics model for noninvasive detection of the physiological and pathological changes in the coronary artery, thus providing a new noninvasive modeling index. Meanwhile, without the step of simulating maximal blood filling, our new method is closer to the true physiological state during CTA scanning.

We have demonstrated that, compared with using a stenosis diameter≥50% as the diagnostic standard for indicating obstructive coronary artery disease, iFR_CT_ could increase diagnostic specificity fourfold, PPV by 74.38%, and accuracy by 68.17%. The significant increase in specificity will avoid unnecessary invasive examinations and reduce medical costs. Compared with the diagnostic efficacy of iFR_CT_ alone, combining iFR_CT_ with CTA slightly enhances the diagnostic specificity, PPV, and diagnostic accuracy. Although no significant difference was found between the diagnostic efficacy of iFR_CT_ alone and iFR_CT_ combined with CTA, 2 cases classified as false positive by iFR_CT_ were reclassified as true negative using combined diagnosis. This improved the diagnostic performance of coronary CTA for functional ischemia, showing the potential of coronary CTA combined with iFR_CT_ as the gatekeeper for ICA and revascularization therapy.

Beam hardening effects, halo artifacts (blooming), and partial volume effects could lead to overestimation by CTA of the degree of coronary stenosis caused by calcified plaques.^[33,34]^ The current study showed good correlation between iFR_CT_ and FFR (*r* = 0.88, *P* < 0.01) in the significantly calcified plaque group, in which iFR_CT_ demonstrated highly accurate diagnostics and specificity of 94.44% and 100%, respectively. Although our study divided cases into different groups based on the number of calcified plaques in the lesion, the results were similar to previous studies using Agatston score. Miyoshi et al analyzed the data of the Japanese NXT study, and reported that FFR_CT_ still had a high diagnostic efficiency (accuracy of 85% and specificity of 81%) for patients with an Agatston score of 400 to 1000.^[35]^ Norgaard et al analyzed 333 lesions in 214 patients and showed that, compared with CTA, FFR_CT_ had a higher diagnostic efficiency for ischemic stenosis in patients with a high Agatston score (416–3599) (AUC: 0.86 vs 0.72; *P* = 0.09), and in vessels with a high Agatston score (121–1703) (AUC: 0.91 vs 0.71; *P* = 0.004).^[36]^ There are 2 possible reasons for this. First, the cases with severe coronary artery calcification were reconstructed using sharp convolution function reconstruction algorithm, which improved the spatial resolution and the boundary between the vessel lumen and calcified plaques, allowing easier luminal identification in modeling. Second, in addition to correlation with luminal border, hemodynamic simulation of iFR_CT_ also involved other parameters, such as blood pressure, blood viscosity, myocardial mass, and heart rate.^[22]^ This decreased the influence of inaccurate luminal identification on the results, which is unlike the coronary CTA that solely relies on the luminal outline. Such results suggest that iFR_CT_ may be a good auxiliary method for CT in the evaluation of coronary artery stenosis caused by severe calcification. This avoids excluding patients with severe calcification from the indications for CTA examination, providing a new method by which coronary artery disease can be examined without invasive techniques in a high-risk population with severe calcification.

However, the current study has the following limitations: the sample size was relatively small. The proportion of patients with positive FFR was only 36.17%, indicating possible selection bias. Because of the small sample size, our study analyzed diagnosis effectiveness based only on blood vessels rather than patients. Although the iFR_CT_ threshold value for the diagnosis of functional stenosis was provided, data from a larger sample are needed to verify the results. This study included only patients with chronic coronary artery disease without a history of coronary artery surgery and acute myocardial infarction; therefore, the applicability of iFR_CT_ in patients with PCI, bypass surgery, and acute coronary syndrome is unknown. In this study, FFR≤0.80 was specified as the diagnostic standard for functional stenosis, as with most of the similar studies in recent years.^[10,22,32,37]^ But FFR is not only influenced by the severity of luminal stenosis, but also by coronary artery morphology and plaque characteristics.^[38–40]^ Therefore, the diagnosis efficiency of iFR_CT_ for patients with different plaque characteristics and coronary artery morphology needs further validation. The modeling calculation was complicated and time-consuming. Data processing was carried out by independent laboratories, and the simulation and calculation on each patient took about 4 hours, which was affected by the performance of the computer and modeling software used. By upgrading and optimizing equipment and technology, the calculation time is expected to be significantly reduced.

## 5 Conclusions

iFR_CT_ is strongly correlated with invasive FFR, and has a higher accuracy, specificity, and PPV than CTA in the diagnosis of functional coronary artery stenosis. Combining with iFR_CT_ can improve the diagnostic accuracy of coronary CTA in the diagnosis of functional stenosis, which also has a good diagnostic ability for coronary stenosis caused by lesions with severe calcification. iFR_CT_ is expected to become a noninvasive, adenosine-independent physiologic index for the diagnosis of ischemia-caused coronary stenosis based on resting state coronary CTA; however, additional studies are needed to determine whether iFR_CT_ has the same diagnostic accuracy as FFR_CT_ for the detection of ischemia-causing coronary stenosis.

## Acknowledgments

The authors thank Dr Quanmin Jing (Department of Cardiology, General Hospital of Shenyang Military Command, Shenyang, China), Dr Benqiang Yang (Department of Radiology, General Hospital of Shenyang Military Command, Shenyang, China), and Dr Haishan Zhang(Department of Cardiology, the First Affiliated Hospital of China Medical University, Shenyang, China) for their help in collecting patient.
